# Chloroplast Genome of Novel Rice Germplasm Identified in Northern Australia

**DOI:** 10.1007/s12042-014-9142-8

**Published:** 2014-10-30

**Authors:** Marta Brozynska, Ernnie Syafika Omar, Agnelo Furtado, Darren Crayn, Bryan Simon, Ryuji Ishikawa, Robert James Henry

**Affiliations:** 1 Queensland Alliance for Agriculture and Food Innovation, University of Queensland, Brisbane, Australia; 2 Australian Tropical Herbarium, James Cook University, Cairns, Australia; 3 Faculty of Agriculture and Life Science, Hirosaki University, Hirosaki, Aomori Japan

**Keywords:** Wild rice, *Oryza*, Chloroplast sequence, Phylogeny

## Abstract

Rice (*Oryza sativa* L.) was probably domesticated from *O. rufipogon* in Asia in the last 10,000 years. Relatives of cultivated rice (A genome species of *Oryza*) are found in South America, Africa, Australia and Asia. These A genome species are the close relatives of cultivated rice and represent the effective gene pool for rice improvement. Members of this group in Northern Australia include, an annual species, *O. meridionalis*, and two recently distinguished perennial taxa, to one of which the name *O. rufipogon* has been applied and the other a perennial form of *O. meridionalis*. Comparison of whole chloroplast genome sequences of these taxa has now been used to determine the relationships between the wild taxa and cultivated rice. The chloroplast genomes of the perennials were both found to be distinguished from *O. rufipogon* from Asia by 124 or 125 variations and were distinguished from each other by 53 variations. These populations have remained isolated from the overwhelming genetic impact of the large domesticated rice populations in Asia and may be unique descendants of the gene pool from which domesticated rice arose. The conservation of this wild genetic resource may be critical for global food security.

## Introduction

The evolution of rice within the grasses has been the subject of considerable research effort. The grasses are considered by some to be of Gondwanan origin (Bouchenak-Khelladi et al. 2010, although there is still a body of thought that attributes a later origin and their distribution to long-distance dispersal. Kellogg (2009) uses this line of reasoning to date the rice tribe back to the start of the Miocene at 20.5 Mya. Earlier publications (Clifford and Simon 1981; Simon and Jacobs 1990) have proposed a Gondwanan origin of the grasses, particularly with reference to Australia. Recent fossil evidence, based on examination of cuticles with silica bodies (phytoliths) suggests that the Oryzeae tribe may have been distinct as early as the Late Cretaceous at 65 Mya (Prasad et al. 2011), but Stevens (2014) questions the correct identity of these fossils and the major conflict with the data assembled from chloroplast and nuclear data. The phylogenetic relationships between *Oryza* species have been widely analysed and the origin and phylogenetic tree for rice genomes types, both diploids (AA, BB, CC, EE, FF and GG) and allotetraploids (BBCC, CCDD and HHJJ, HHKK) is well determined (Ammiraju et al. 2010; Ge et al. 1999; Lu et al. 2009). However, the more recent origin of the *Oryza* genus and the A genome clade of *Oryza* species from which rice was domesticated has not been well defined. Despite numerous attempts to resolve diversification of the A genome species (e.g. Zhu and Ge (2005); Kwon et al. (2006); Duan et al. (2007), and Zou et al. (2008)), no conclusive phylogenetic relationship of these species have been generated from those studies, nor has the method or timing of these events been defined. Regarding distribution, if the Gondwanan explanation is invoked, the A genome species have had their present distribution for a much longer time than if long-distance dispersal is used to explain how very similar species occur in four continents.

**Fig. 1:**
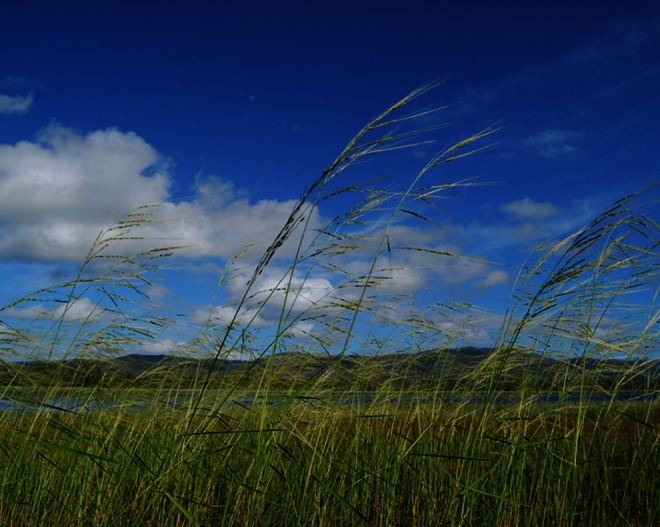
Australian perennial wild rice (Taxon A) growing in its natural habitat in Northern Queensland, Australia. Open panicles and anther length (>3–7.4 mm) affirm its morphological resemblance to *Oryza rufipogon*

The greatest concentration of diversity in *Oryza* is found in South East Asia and Northern Australia (Vaughan et al. 2008). Both annual and perennial species of wild rice, belonging to the A genome type, have been identified (Vaughan et al. 2008). In Asia, the perennial species is referred to as *O. rufipogon* while the annual form is referred to as a distinct species *O. nivara* (Oka 1974; Sharma 2003). Four *Oryza* species are currently recognized from Northern Australia with two of these, one an annual and the other a perennial, belonging to the A genome clade (Henry et al. 2010). The perennial wild rice in Australia is referred to as *O. rufipogon* while the annual species is known as *O. meridionalis*. Waters et al. (2012) used whole chloroplast genome sequencing to show that the Australian and Asian wild rices, including both the annual and perennials, were distinct from each other. However, only one accession of the Australian perennial wild rice, referred to as *O. rufipogon*, was analysed by Waters et al. (2012). Recent studies by Sotowa et al. (2013) identified two distinct types within the Australian perennial wild rice populations, suggesting that the Australian populations that had been identified as *O. rufipogon* consist of two different types of perennial wild rices which may be distinct species. One of the perennial populations was referred to as the m-type as it is morphologically similar to *O. meridionalis* (closed panicles and short anthers) and shared two loci in the chloroplast genome. This population, here referred to as Taxon B, may have a common ancestor with *O. meridionalis*. Chloroplast gene sequence data (Sotowa et al. 2013) suggested that this was the type included in the study by Waters et al. (2012) then referred to as Australian *O. rufipogon*. Sotowa et al. (2013) combined molecular and morphological evidence to suggest that this perennial was a new taxon distinct from both the annual *O. meridionalis* and from the Asian *O. rufipogon*. The other Australian perennial type wild rice population, referred to as the r-type (Sotowa et al. 2013), was morphologically similar to *O. rufipogon* suggesting a possible common origin (Fig. 1). We now report the complete chloroplast genome sequence for an accession of this second *O. rufipogon*-like Australian wild rice (Taxon A) and comparison with the complete chloroplast sequence of other wild and domesticated rices. This study aims to further clarify the relationships between the Australain and Asian wild *Oryza* from the A genome clade by comparing the whole chloroplast genomes of all of the known taxa for the first time.

## Materials and Methods

### Plant Material

The wild rice plant used in the study (Species A) was collected from a population of perennial wild rice at Abattoir Swamp Environmental Park in North Queensland, Australia (S16.38.085, E145.19.366). This group of wild rice plants, illustrated in Fig. 1, was described by Sotowa et al. (2013) as *Oryza rufipogon*-like (r-type) and was found at the Jpn1 site. In this study it is referred as “wild rice Taxon A”.

### DNA Extraction, Sequencing and Data Analysis

The chloroplast genome of a wild rice Species A collected from northern Australia was sequenced using next generation sequencing of total DNA. DNA from leaf tissue of a wild rice plant was extracted using a modification (Carroll et al. 1995) of the CTAB method (Bernatzky and Tanksley 1986) as described by (Furtado 2014). Illumina HiSeq2000 (Illumina, San Diego, CA, USA) and Ion Torrent (PGM, #318 chip) sequencing platforms were used for shotgun sequencing of total genomic DNA from the sample. A consensus sequence of the wild rice Species A chloroplast genome was created and validated using the following approaches: (1) Illumina and Ion Torrent read mapping to the reference chloroplast sequence of *Oryza sativa japonica* cv. Nipponbare, (2) *de novo* Illumina reads assembly and subsequent contig mapping to the reference, and (3) reference-assisted reads assembly from Ion Torrent platform followed by ordering the contigs based on the *O. sativa* reference sequence. All the Illumina analyses were performed on CLC Genomics Workbench 6.0 (www.clcbio.com/) and Ion Torrent reads were alternatively imported to Torrent Suite Software version 3.6. Default programmes from both software platforms were used for quality analysis, read trimming, read mapping and *de novo* or reference-based read assembly.

### Functional Annotation

Wild rice Taxon A chloroplast genome annotation was performed using the Bacterial Genome Annotation system BG7 (Pareja-Tobes et al. 2012) followed by manual curation of exon/intron boundaries and start/stop codons.

The full circular chloroplast gene map was visualised using the OrganellarGenomeDRAW tool (Lohse et al. 2013).

### Phylogenetic Analysis

Eight fully sequenced chloroplast genomes of diploid rice species were used to perform a phylogenetic study (Table 1). All of the species, but one, were A genome rice relatives. *Oryza australiensis*, belonging to EE rice genome group, was included as an outgroup as this species is less closely related to the other *Oryza* genus species analysed in this study.

**Table 1:** Summary of rice chloroplast sequences used in phylogenetic analysis and comparative study: their genome group, full chloroplast length, length without one inverted repeat (IR) sequence and GenBank accession number

Rice species	Genome group	Full length chloroplast sequence (bp)	Sequence length without IR (bp)	Accession number
Taxon A	AA	134,557	113,754	KF428978
*O. sativa japonica*	AA	134,551	113,749	GU592207
*O. sativa indica*	AA	134,496	113,698	AY522329
*O. meridionalis*	AA	134,558	113,755	JN005831
Taxon B	AA	134,557	113,754	JN005833
*O. rufipogon* (Asia)	AA	134,544	113,743	JN005832
*O. nivara*	AA	134,494	113,692	AP006728
*O. australiensis*	EE	134,549	113,749	GU592209

*IR*, Inverted repeat

The consensus chloroplast sequence of the wild rice Species A was aligned with other publicly available chloroplast genomes of species from the genus *Oryza* (GenBank, http://www.ncbi.nlm.nih.gov/). The following sequences were used in the study: *O. sativa* spp. *japonica* cv. Nipponbare, *O. sativa indica* isolate 93–11, Australian perennial sample used by Waters et al. (2012) and referred therein as *O. rufipogon* (referred in the present study as “wild rice Species B” due to its similarity to populations relating to the *O. meridionalis*-like perennials from the Jpn2 site in Sotowa et al. (2013)), Asian *O. rufipogon*, *O. meridionalis*, *O. nivara*, and *O. australiensis*. A summary of the genome groups, sequence lengths and GenBank accession numbers are shown in Table 1. The multiple genome alignment was conducted using Mauve 2.3.1. software and the progressive Mauve algorithm (Darling et al. 2010) with default parameters. Prior to the analysis one copy of the inverted repeat (IR) sequence was deleted from the chloroplast sequence of each of the genomes. The length of the IR ranged between 20,792 and 20,803 bp depending on the species.

The phylogenetic tree reconstruction for chloroplast genome alignment was performed using three distinct methods: maximum parsimony (MP), maximum likelihood (ML) and Bayesian posterior probability. Selection of the best-fit model of nucleotide substitution was conducted using jModelTest2 (Darriba et al. 2012) software and Akaike information criterion. The model chosen for maximum likelihood calculation was 012010 + I + G + F (I = 0.8990). MP and ML analyses were completed in PAUP* 4.0 software package (Swofford 2003) choosing a heuristic search for finding the optimal tree, the random stepwise addition procedure for obtaining a starting tree and with the tree bisection-reconnection (TBR) algorithm for branch-swapping. 200 random-addition sequence replications were performed, as well as 2,000 bootstrap pseudoreplications to measure group support (frequency of occurrence). Gaps were considered as missing data. All characters were treated as unordered and weighted equally.

Bayesian analysis was performed using MrBayes through the Geneious 6.1.2 software platform (www.geneious.com/). The evolutionary model used was the General Time Reversible Model with gamma-shaped among-site rate variation with an estimated proportion of invariable sites (GTR + I + G; I = 0.8990). The branch length prior was set to exponential with parameter 10.0. Two independent and simultaneous analyses starting from diverse random trees were performed. Monte Carlo Markov Chains (MCMC) were run for 1 × 10^6^ generations, with chains sampled every 200 generations, followed by burn-in of 1 × 10^5^ MCMC. Three heated chains and one cold chain were used with the heating coefficient of 0.2 (by default). Consensus nodal support was assessed by posterior probability distribution.

All trees were rooted using the outgroup method.

### Distances Between Rice Chloroplast Genomes and Comparative Chloroplast Genomics

Each of the full length rice chloroplast genomes was imported into the Geneious 6.1.2 software platform and the sequences were aligned using the alignment tool available in the package, followed by variant analysis using the variant/SNP detection tool. In order to create a distance matrix for chloroplast genomes all the variants, as well as the differences (i.e. number of non-identical bases) were counted. The variants between *Oryza* Taxon A and other rice chloroplast were counted once, regardless of their length.

## Results

### Chloroplast Genome Features of Wild Rice Taxon A

**Fig. 2:**
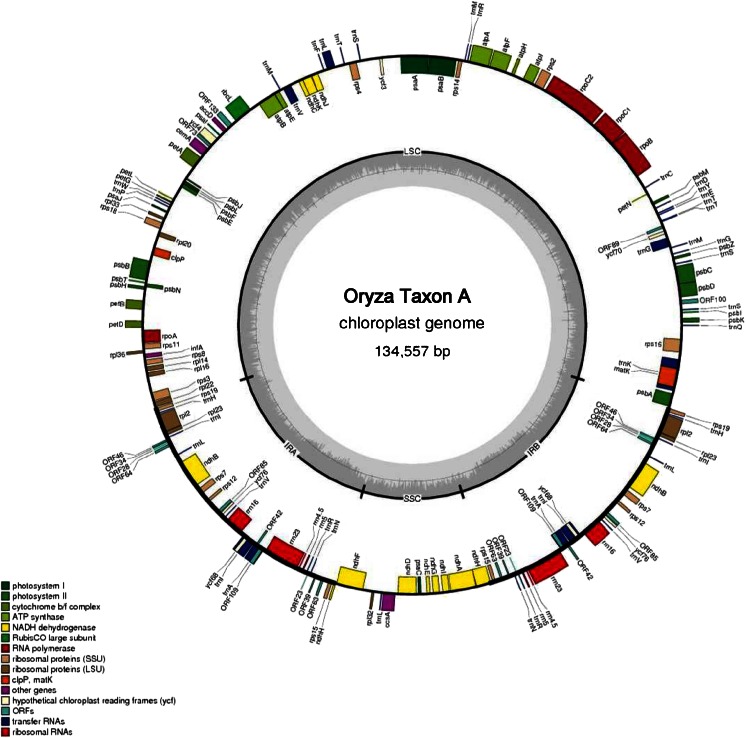
A chloroplast genome map of an Australian perennial wild rice (Species A). The *inner circle* indicates the boundaries between large single copy (LSC), inverted repeats (IRA and IRB) and small single copy (SSC). The dashed area shows the GC content of the genome and marks the 50 % threshold with additional *grey line* inside. Genes are transcribed either clockwise (those found outside the outer circle) or counter clockwise (inside the outer circle). Features which belong to diverse functional groups are marked with different colours

The plastid genome of Taxon A consists of the four typical components found in angiosperms, i.e. two inverted repeats regions, IR_A_ and IR_B_ (both 20,803 bp in length), a large single copy (LSC) (80,604 bp) and a small single copy (SSC) region (12,347 bp) (Fig. 2). Overall 162 genes were found in the chloroplast genome, including 114 protein-coding genes, 40 transfer RNAs and eight ribosomal RNAs. Twenty CDS and eight tRNAs were duplicated owing to the location in each of the inverted repeats. rRNAs genes were located in the IR forming two operons of 23, 4.5 and 5S, and two separate 16S subunits. A total of 97 genes were single copy in the wild rice Species A chloroplast genome (75 protein-coding genes and 24 tRNAs). A functional description and gene visualisation is presented in Fig. 2. Eleven genes contained one intron: 6 tRNA genes (*tRNA*-*Lys*, *tRNA*-*Gly*, *tRNA*-*Leu*, *tRNA*-*Val*, *tRNA*-*Ile* and *tRNA*-*Ala*) and five protein-coding genes (*rps16*, *atpF*, *rpl2*, *ndhB and ndhA*). The genome is AT-rich with an AT content of 61 %. Coding sequences occupy 58.29 % of the genome, comprising protein coding regions (49.24 %), rRNA (6.83 %) and tRNA (2.22 %). Non-coding sequences contain intergenic regions and introns which represent 33.01 and 8.70 % of the chloroplast genome, respectively (Fig. 4b).

### Phylogenetic Analysis

**Fig. 3:**
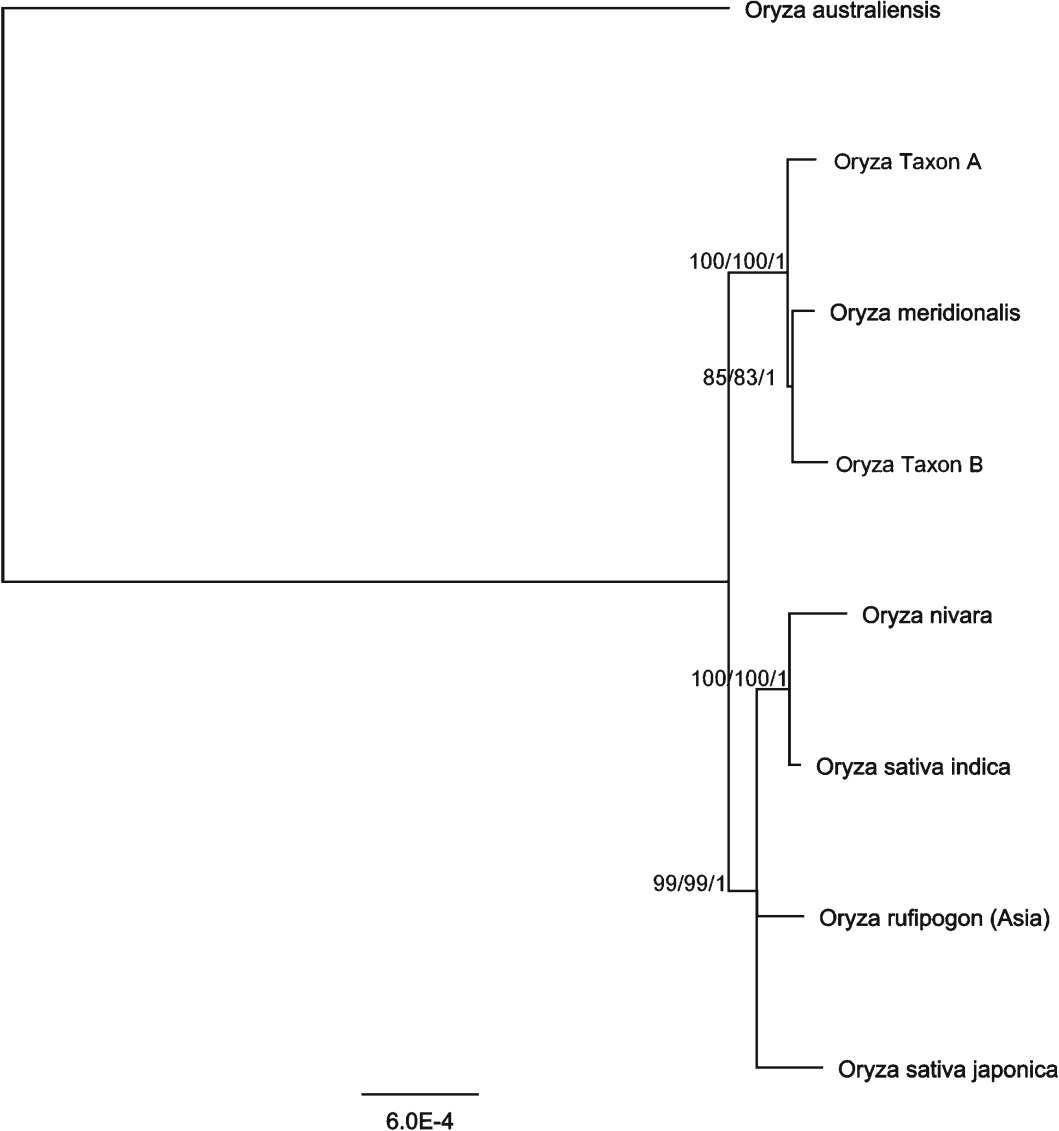
Phylogenetic relationships between chloroplast genomes of *Oryza* species. Nodal support is presented as precent bootstrap in MP/precent bootstrap in ML/Bayesian posterior probability. *Scale bar* is the number of substitutions per site

The multiple genome alignment of rice chloroplast sequences was 113,960 bp in length. One of the inverted repeats was excluded prior to phylogenetic analysis due to the identical sequence of these repeats. This avoids any parsimony informative sites in the inverted repeat regions being weighted with twice the value relative to other informative sites in the alignment. Of the total number of bases which were subjected to maximum parsimony (MP) analysis, 112,969 were constant, 903 were variable and parsimony uninformative and the number of parsimony informative characters was 88. The most parsimonious tree was obtained after 798 rearrangements during a heuristic search and was 1014 in length with consistency index CI = 0.98 and retention index RI = 0.89. The CI excluding uninformative characters was 0.84. The score for the best tree (−lnL) under maximum likelihood (ML) criterion was 162388.46454 after 142 rearrangements. Bootstrap nodal support calculated from both, MP and ML, was strong (>99.2 %) except for the node of Australian wild rice Species B and *O. meridionalis* where the support was slightly lower (83 % in ML and 85 % in MP). Posterior probabilities of the tree resulted from Bayesian inference were all 100 %. The optimal trees found by all three phylogenetic methods were consistent and the final tree is presented in Fig. 3.

### Distances Between Rice Chloroplast Genomes

Distances between the individual chloroplast genomes from all of the species studied were represented as the number of variants between each of them (variant represented a SNP or multi-nucleotide variants (MNV) regardless of its length). Additionally the nucleotide differences in the sequences (i.e. number of bases which are not identical) were also determined (Table 2).

**Table 2:**
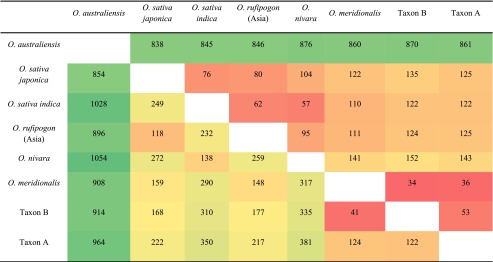
Distance matrix of rice species chloroplast genomes

The top panel represents number of variants between species (a variant corresponds to a single nucleotide- or a multi nucleotide-variant at any given position). The bottom panel represents number of nucleotide differences in the sequences (numbers of non-identical bases). The heatmap corresponds to individual values in a matrix as colours: red cells indicate close distance; orange, yellow and green cells show gradually greater distances

The lowest number of variants within the Australian clade, and also within all the species studied, was found between wild rice Taxon B and *O. meridionalis* (34 variants). Interestingly, there were more variants between wild rice Species B and wild rice Species A (53 variants) than between *O. meridionalis* and each of these perennial species (34 and 36 variants, respectively). The Australian wild rice Species A analysed in the present study had a considerably lower number of differences in comparison with the other Australian A genome species than with the Asian species. Within the Asian clade all of the species’ chloroplast genomes had the lowest number of polymorphisms with *O.sativa* spp. *indica* in the following order: *O. nivara* (57), *O. rufipogon* (62) and *O. sativa japonica* (76). As expected, considerably greater distances were present between *O. australiensis* and all other rice species.

### Comparative Chloroplast Genomics

**Fig. 4:**
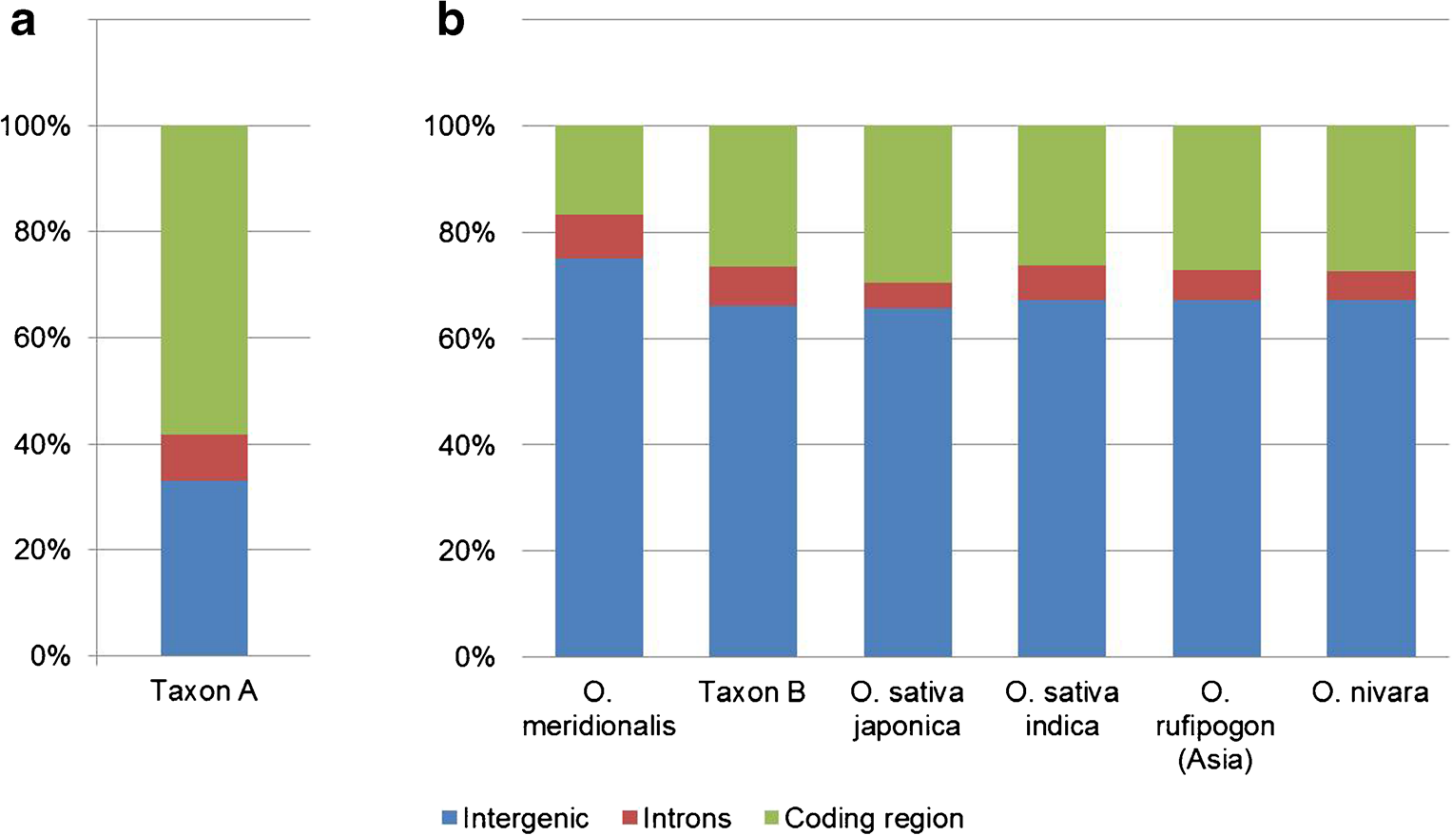
Analysis of coding and non-coding regions in chloroplast genomes. (**a**) Distribution of sequence types in *Oryza* Taxon A and (**b**) proportion of variants relative to *Oryza* Taxon A found in each of these regions

Table 3 shows the number and types of variants in the chloroplast genomes of the Australian wild rice and other rice species used in this study. The most abundant variation types in all species were SNPs. The chloroplast genome is mainly composed of coding region followed by intergenic region while a small component is comprised of introns (Fig. 4a). However the distribution of variants as a proportion is mainly in the intergenic regions (66–75 % variants) followed by coding region (17–30 % variants) and a small component in introns (5–8 % variants) (Fig. 4b). The majority of polymorphisms were found in the intergenic regions which occupy only 33.01 % of the chloroplast genome, almost two times less than the coding sequences (58.29 %).

**Table 3:** Total number and type of sequence variants in rice species chloroplast genomes in comparison with Taxon A

Rice species	Variants number	Deletions	Insertions	MNV	SNPs (Ts/Tv)
*O. meridionalis*	36	4	10	3	19 (8/11)
Taxon B	53	6	9	5	33 (16/17)
*O. sativa indica*	122	18	17	10	77 (39/38)
*O. rufipogon* (Asia)	125	18	14	7	86 (40/46)
*O. sativa japonica*	125	14	17	4	90 (47/43)
*O. nivara*	143	21	20	10	92 (46/46)
*O. australiensis*	861	14	16	20	811 (518/293)

*MNV*, Multi-nucleotide variant; *Ts* and *Tv*, Transitions and transversions, respectively

The SNPs found in coding regions of chloroplast genomes were analysed based on their effects on protein sequences. Synonymous and non-synonymous substitutions, as well as frame shifts were discovered among genomes (Table 4). In order to acquire a better understanding of the variation or differences present in protein coding regions between the Australian and Asian *Oryza* clades, as well as the unique occurrences in the Australian wild rice, the differences were grouped together based on their origin (Table 5). Sixteen SNPs and two indels were found to be specific to either the Australian or Asian clade. Six out of them resulted in amino acid substitutions. Four SNPs were unique to the wild rice (Taxon A) and one of them caused an amino acid substitution of asparagine to serine in *rpoC1* gene (DNA-directed RNA polymerase subunit beta’). All of the proteins with variants were blasted against non-redundant protein sequences (nr) on the NCBI database (http://blast.ncbi.nlm.nih.gov/) to investigate the uniqueness of the substitutions in the chloroplasts of Asian and Australian rices, as well as in other plants. Among the substitutions analysed, two were found to be exclusive to the Australian clade, one to the Asian, and one in Taxon A and two were found in numerous plant species (Table 6).

**Table 4:** SNPs and frame-shift events in coding region of the chloroplast genomes of rice species in comparison with Taxon A

Rice species	Synonymous SNPs	Non-synonymous SNPs	Frameshifts
*O. meridionalis*	5	1	0
Taxon B	6	7	0
*O. sativa japonica*	22	11	4
*O. sativa indica*	17	9	4
*O. rufipogon* (Asia)	16	15	3
*O. nivara*	22	11	3

*SNP*, Single nucleotide polymorphism

**Table 5:** Polymorphisms in chloroplast coding regions found to be specific to Australian or Asian clades or Taxon A

Position	Australian clade	Asian clade	Taxon A	Gene	Protein effect
**LSC**					
448	G	A	G	psbA	*
817	G	A	G	psbA	*
2,220	G	T	G	matK	Ala → Glu
3,067	G	T	G	matK	Leu → Ile
4,546	A	T	A	rps16	*
9,321	G	G	***A***	psbD	*
24,164	A	A	***G***	rpoC1	Asn → Ser
24,742	T	G	T	rpoC2	His → Gln
25,000	G	A	G	rpoC2	*
27,949	C	A	C	rpoC2	*
29,885	T	C	T	rps2	*
55,325	G	A	G	rbcL	*
**IR**_**A**_					
90,581	G	T	G	ycf76	*
93,538	C	del	C	ycf68	frameshift
**SSC**					
104,506	T	T	***C***	rpl32	*
105,927	C	A	C	ccsA	Ser → Tyr
106,567	G	T	G	ndhD	Ala → Glu
106,710	G	T	G	ndhD	*
110,849	G	A	G	ndhA	*
113,255	G	G	***A***	ndhH	*
**IR**_**B**_					
121,627	G	del	G	ycf68	frameshift
124,581	C	A	C	ycf76	*

Positions refer to the positions in Taxon A chloroplast genome. Amino acid changes refer to a change with respect to Australian clade and Taxon A with the exception of position 24,164 (Ser is exclusive for Taxon A). Australian clade includes: *O. meridionalis* and Taxon B; Asian clade includes: *O. sativa japonica* and *indica*, *O. rufipogon* (Asian origin) and *O. nivara*. Nucleotides marked in bold and italics are found to be unique to Taxon A. LSC: long single copy; SSC: short single copy; IR_A_ and IR_B_: inverted repeats; del: deletion, *: synonymous SNP

**Table 6:** Predicted amino acid substitutions in chloroplast encoded proteins between Australian and Asian clades of *Oryza*

Position	Australian clade	Asian clade	Taxon A	Gene	Uniqueness of substitution
46	Leu	Ile	Leu	*matK*	Leu exclusive for Australian clade
328	Ala	Glu	Ala	*matK*	known
567	Asn	Asn	Ser	*rpoC1*	Ser exclusive for Taxon A
10	His	Gln	His	*rpoC2*	Gln exclusive for Asian clade
224	Ser	Tyr	Ser	*ccsA*	Ser exclusive for Australian clade
452	Ala	Glu	Ala	*ndhD*	known

Positions refer to the amino acids positions in given proteins from Taxon A; known – given substitution was found among other genes from chloroplast genomes of numerous plant species; exclusive for Australian/Asian clade – given substitution was found only in Australian or Asian clades, respectively; exclusive for Taxon A – given substitution was found only in Australian wild rice (Taxon A)

There was also a frame-shift found in one of the chloroplast encoded proteins of unknown function, *Ycf68*, which is duplicated in the chloroplast genome due to its location in the inverted repeats (the frameshift was present in both copies as these regions are perfect repeats). The frame-shift is produced by a single base insertion in the Australian chloroplast genomes that causes an early stop codon and protein truncation (in Asian genomes it is 133 amino acids in length, in Australian 101 amino acids) (data not shown). Despite the premature termination of the gene *ycf68* the functional protein domain (which lies in N-terminal end) is still present in the protein sequence (Uncharacterised protein family Ycf68, InterPro: IPR022546, http://www.ebi.ac.uk/interpro/). Also proteins of similar length were found in other plant species, e.g. *Triticum monococcum* subsp. *aegilopoides* (GenBank accession AGP51191) and *Setaria italica* (Doust et al. 2009).

The chloroplast genome lengths vary in the *Oryza* genus between 134,494 bp (*O. nivara*) and 134,558 bp (*O. meridionalis*). The length of the wild rice Taxon A chloroplast genome was 134,557 bp which corresponds with usual *Oryza* chloroplast length and with standard sizes of plastid genomes described so far (120–160 kbp (Green 2011)). The chloroplast genome of the Australian wild rice Taxon A is AT rich as reported for other angiosperms (Raubeson et al. 2007).

## Discussion

Long indels found between *O. nivara* and *O. sativa indica* and the other rice species (data not shown) affected the distance analysis among rice chloroplast genomes increasing the number of different nucleotides and consequently the distance from other rice relatives (Table 2). However, the presence of indels did not affect the phylogeny which placed these two species together within the Asian clade, as has been reported by others (Huang et al. 2012), and indicates that it is very likely that each of those indels was one separate evolutionary event.

The most dominant cultivated rice species (*Oryza sativa*) belongs to the A genome group, and was most likely domesticated from wild populations of *O. rufipogon* in Asia. The A genome group is the most recently diverged group in the *Oryza* genus (Vaughan 1989; Ge et al. 2001) and is comprised of eight diploid species, with annual and perennial types, and distributed worldwide including Australia (Vaughan 1989). The Australian perennial wild rice of the A genome type and generally referred to as Australian *O. rufipogon* (Vaughan et al. 2008; Henry et al. 2010), has recently been shown to be comprising of at least two types, the m-type and the r-type based on the analysis of loci in the nuclear genome (Sotowa et al. 2013). In addition, Sotowa et al. (2013) examined many accessions of these taxa and reported that all wild type perennials found in Australia had meridionalis-type plastid genomes on the basis of analysis of two loci within the chloroplast. In our study, the best phylogenetic tree obtained confirms the distinctness of Australian and Asian rice relatives as reported by Waters et al. (2012). Moreover the phylogenetic analysis placed the perennial wild rice Taxon A within the Australian clade and also supported earlier findings about perennial wild rice Species B (Waters et al. 2012). Most importantly this study shows for the first time the distinctness of the Australian Taxon A from *O. rufipogon*, despite superficial morphological resemblance the chloroplast genome shows 125 variations. This indicates that the genetic difference between Taxon A and *O. rufipogon* (125) is comparable with that between Taxon A and *O. sativa japaonica* (125) and that between *O. rufipogon* and *O. sativa japonica* (118). This perspective suggests that the Australian perennial, *Oryza* Taxon A, is a similar genetic distance from both domesticated Asian rice (*O. sativa*) and the wild Asian perennial rice (*O. rufipogon*) and that this is a similar genetic distance to that found between the wild and domesticated Asian species. The divergence between *O. rufipogon* and *O. sativa* was probably driven by human selection in the last 10,000 years while the divergence of *O. rufipogon* in Asia and *Oryza* Taxon A in Australia was likely to have happened over a much longer period and been driven by allopatric evolution.

The Australian wild rice species previously reported (*O. meridionalis* and the Australian *O. rufipogon* referred here as wild rice Taxon B) were found to be more closely related to each other than the other wild relative (Taxon A) studied here. Based on the distance analysis it can be concluded that rice species within the Australian clade are generally more closely related to each other than the species within the Asian clade which is also reflected in the phylogenetic tree (Fig. 3). The number of differences in the chloroplast between *O. nivara* and *O. sativa indica* (138), and *O. rufipogon* and *O. sativa japonica* (118) is similar to the distance between *O. meridionalis* and *Oryza* Taxon A (124). Based on chloroplast analysis, the relationship between the two morphologically distinct Australian perennial wild *Oryza* species and *O. meridionalis* was relatively close. Both are more closely related to *O. meridionalis* than the two cultivated subspecies of rice *O. sativa*, subsp. *japonica* and subsp. *indica* are to one another (Table 2). The chloroplast genome sequence of *Oryza* Species A confirms that it is distinct from *O. rufipogon*, *O. meridionalis* and *Oryza* Taxon B.

Nuclear genomes not analysed here may provide further information on the relationships between these species. Sotowa et al. 2013 analysed INDEL and SSR markers in the nuclear genome of other wild rice perennial accessions with the morphology of Species A collected from the same site (Jpn1 site) revealing some individuals to be closer to *O. meridionalis* and others to *O. rufipogon* at the loci tested. Analysis of the complete nuclear genomes may provide further information on the relationships between these Australian perennial accessions and their relatedness to cultivated rice.

The chloroplast genome sequences used here provide a guide to the relationships between the taxa but analysis of the nuclear genomes could result in greater insights into the evolution of the A genome clade of *Oryza*. Earlier studies explored some possible relationships between these taxa on a morphological basis (Sotowa et al. 2013). Despite some superficial morphological similarities, these two Australian wild rice perennial lineages, the r-type (Taxon A) and the m-type (Taxon B), have now been characterised as considerably divergent from both, *O. rufipogon* and *O. meridionalis*, and especially from one another at organellar, nuclear and morphological levels.

This discovery adds further weight to present a case for South East Asia and Northern Australia being considered as the centre of origin for the A genome. While the tribe is possibly of Gondwanan origin the divergence of the A genome species is likely to be a relatively recent event involving long distance dispersal throughout the tropical world. More collections are required to determine the distribution of this species and to define the genetic variation within the population. This becomes more urgent with the prospect of agricultural expansion in northern Australia and with rice cultivation becoming more likely. The wild rice populations in Australia have to date been isolated from the impact of genetic contamination by variants of cultivated rice as has been reported to have occurred widely in Asia with *O. rufipogon*. Pressure for more rice to satisfy food security could see extensive rice cultivation occur in Northern Australia. The conservation of this wild genetic resource may require efforts in both *ex situ* and *in situ* conservation. The perennial A genome rice species from northern Australia are expected to be a valuable new genetic resource for rice improvement, with potential to contribute novel disease resistances, environmental stress tolerances and possible nutritional value.
